# How left-behind experience moderates the impact of impulsivity on non-suicidal self-injury among Chinese senior high school students: the mediating role of self-control

**DOI:** 10.3389/fpubh.2026.1813662

**Published:** 2026-07-24

**Authors:** Weixing Zou, Lingping Xie, Guoyan Liu

**Affiliations:** 1 School of Education Science, Minzu Normal University of Xingyi, Xingyi, China; 2 School of Management, Zunyi Medical University, Zunyi, China

**Keywords:** high school students, impulsivity, left-behind experience, non-suicidal self-injury, self-control

## Abstract

**Background:**

Prior research has identified a range of risk factors for and functions of non-suicidal self-injury (NSSI), of which two broad classes are most prominent: adverse early experiences and individual susceptibility. Much less is known, however, about how these two classes of factors operate together within an integrated mechanism. We therefore examined whether self-control mediates the relationship between impulsivity and NSSI in senior high school students, and whether an early left-behind experience moderates this mediated pathway.

**Methods:**

Questionnaires measuring impulsivity, self-control, and NSSI were administered to senior high school students in Guizhou Province, China. A total of 534 valid responses (Mage = 17.26 years, SD = 1.30; 57.62% girls) were analysed.

**Results:**

Impulsivity was positively associated with NSSI, and self-control significantly mediated this association, accounting for a substantial proportion of the indirect effect. Left-behind experience further moderated the mediation pathway: among students with a left-behind experience, the indirect effect of impulsivity on NSSI via self-control was significant, whereas the direct effect was not; among students without a left-behind experience, the opposite pattern was observed. Because the proposed model explained only a modest proportion of the variance in NSSI, these results are best regarded as identifying one theoretically grounded pathway to NSSI rather than as a complete account of how NSSI develops.

**Conclusion:**

Impulsivity is an important correlate of NSSI in senior high school students, and its association with NSSI appears to operate largely through self-control. Moreover, the pathway to NSSI differs markedly between students with and without a left-behind experience. These findings provide an empirical basis for designing more targeted NSSI interventions.

## Introduction

### NSSI: prevalence and an integrated risk framework

Non-suicidal self-injury (NSSI) refers to deliberate, repeated harm to one’s own body without suicidal intent (1). It frequently co-occurs with psychiatric conditions such as eating disorders, bipolar disorder, and borderline personality disorder (2), increases the risk of future suicide attempts (3), and is listed in the Diagnostic and Statistical Manual of Mental Disorders (DSM-5) as a “condition requiring further study” (4).

Recent international and Chinese evidence underscores how pressing this issue has become. A 2022 systematic review and meta-analysis showed that self-injurious thoughts and behaviours in children are far more prevalent than previously assumed and are robustly linked to psychiatric and family-context correlates (5). Within the Chinese context, a meta-analysis of 59 studies (*n* = 192,546 adolescents) reported a pooled NSSI prevalence of 22.5%, with depressive symptoms, negative life events and impulsive tendencies among the strongest correlates (6). These figures position NSSI as a major public health concern in Chinese adolescents and motivate a closer examination of how dispositional, self-regulatory, and family-environmental factors jointly shape its occurrence.

NSSI is especially common and clinically significant during adolescence. International estimates place its prevalence at around 17% (7), and a pooled estimate among Chinese middle-school students is approximately 22.4% (8), figures that converge with the meta-analytic evidence cited above. NSSI typically first emerges in early adolescence (9), at around 12 to 14 years of age (10, 11); episodes last on average about 2 years (12) and tend to peak in late adolescence and early adulthood before gradually declining (13). Late adolescence is therefore a critical window for understanding why some young people develop NSSI while others do not.

Risk factors for NSSI are commonly grouped into two broad categories: adverse early experiences (most prominently abuse and neglect, but also separation from or loss of important caregivers) and individual susceptibility (14). Individual susceptibility, in turn, has been linked to three components: emotional dysregulation (a state-level process active during self-injury), impulsivity (a personality-level disposition), and biological factors (14). This person-by-environment framing organises the present study: we examine one dispositional susceptibility factor (impulsivity), one self-regulatory resource that may channel its effect (self-control), and one early-experience factor that may shape this mechanism (left-behind experience).

### Impulsivity as a personality risk factor for NSSI

Personality comprises the characteristic patterns of thought and behaviour that shape how a person reacts emotionally to, and copes with, events, and certain personality profiles confer greater vulnerability to NSSI. Among the Big Five traits, neuroticism shows the most consistent and strongest association with NSSI (15), whereas findings for extraversion, agreeableness, conscientiousness, and openness remain inconsistent (16–18)—possibly because of differences in measurement instruments, or because the Big Five taxonomy is not ideally suited to NSSI research. In particular, impulsivity is not adequately captured within the Big Five framework (19), yet it has been singled out as theoretically central to NSSI. The present study therefore focuses on impulsivity as a single, theoretically motivated personality predictor of NSSI in Chinese senior high school students. Accordingly, we propose Hypothesis 1 (H1): impulsivity positively predicts NSSI among senior high school students.

Impulsivity is a multifaceted personality trait, typically defined as a tendency to react rapidly to internal or external stimuli without adequate planning, information processing, or regard for the negative consequences for oneself or others (20). It has been identified as a core NSSI risk factor (21) and is explicitly represented as an individual-susceptibility component in Nock’s integrated theoretical model of NSSI (14). The evidence is, however, method-dependent: self-report studies consistently show higher impulsivity in NSSI than in comparison groups, whereas behavioural-task studies (e.g., impulse-control, behavioural disinhibition, and risky-decision paradigms) often find no group difference in adults or adolescents (21–23). Notably, this question has rarely been examined in Chinese adolescents, a gap the present study helps to address.

Consistent with a self-regulatory interpretation, impulsivity is associated with a range of disorders characterised by impaired inhibitory control (including attention-deficit/hyperactivity disorder, schizophrenia, and borderline personality disorder) and with problematic behaviours such as smoking, gambling, and substance abuse (24–26). Highly impulsive adolescents may thus be less able to regulate their thoughts and behaviour, increasing their vulnerability to NSSI.

Barratt distinguished three dimensions of impulsivity: motor impulsivity (acting on the spur of the moment), cognitive impulsivity (not attending to the task at hand), and non-planning impulsivity (lack of forethought) (27). Consistent with the trait’s clinical relevance, impulsive individuals are more likely to attempt suicide (28), and individuals with suicidal ideation are more impulsive than those without (29).

### Self-control as a protective factor and its links to impulsivity

Self-control refers to the capacity to regulate one’s behaviour, emotions, and cognition in accordance with social standards or personal goals (30, 31). From a trait perspective, self-control is the ability to override internal responses and to interrupt or inhibit unwanted behavioural tendencies (32); from a state perspective, it denotes the momentary exercise of control over oneself (33). Individuals high in self-control are better able to regulate and maintain their own psychological state (34).

During the high-school years, self-control is central to students’ psychological, physiological, and behavioural self-regulation (30), and most adolescents who engage in NSSI show impaired emotion regulation (9, 35). This makes self-control a theoretically plausible factor for understanding NSSI in this age group.

Empirically, low self-control predicts increased antisocial and rule-breaking behaviour (36) as well as smoking, drug use, and alcohol abuse (37), and it is associated with a wide range of problematic behaviours including procrastination, impulse buying, addictive behaviours, and offending (38–41). Because these behaviours frequently generate negative emotions such as depression, stress, and guilt (42–44), low self-control may contribute, directly and indirectly, to NSSI. Conversely, higher self-control is associated with greater affective well-being and life satisfaction (45, 46), an association that appears to operate partly through a stronger focus on self-improvement rather than loss avoidance (47); adolescents with poorer self-control may thus be doubly disadvantaged, being both more exposed to problem behaviour and less able to sustain positive emotional states.

The self-control strength model (48) holds that exercising self-control consumes a limited pool of psychological resources; once these are depleted (a state termed ego depletion), subsequent attempts at self-control are more likely to fail. The success of a given self-control effort therefore depends on the resources available at that moment.

Numerous studies have shown that, in a state of ego depletion, individuals who would ordinarily refrain from undesirable behaviour become less able to inhibit impulsive actions, including aggression (49), inappropriate sexual behaviour (50), prejudiced responding (51), overeating (52), excessive drinking (53), and impulse buying (54).

Self-control thus appears to be a key mechanism linking other risk factors to adolescents’ problematic behaviour, and low self-control may be an important contributor to NSSI. This is consistent with the observation that emotional and cognitive regulation functions both as a motivating function of NSSI in Nock’s integrated model (14) and as a defining component of self-control. Yet the direct influence of self-control on NSSI remains under-studied. We therefore propose Hypothesis 2 (H2): self-control negatively predicts NSSI in senior high school students.

Chinese culture, relative to individualist cultures, places strong emphasis on moral norms and social relationships. As self-awareness develops during adolescence, Chinese adolescents may more frequently encounter conflicts between external normative demands and internal needs, and these conflicts may be more intense, placing greater demands on self-control. Where self-control fails, NSSI may serve as one maladaptive response, which may help to explain the relatively high NSSI prevalence reported in this population.

The antecedents of self-control have received comparatively little attention. The available evidence indicates that self-control develops from childhood into adolescence and that more supportive parental relationships and friendships are associated with stronger self-control (55–57); consistent with this, secure (rather than insecure) attachment is linked to better self-control (32). It follows that an unsupportive environment—including being left behind by migrant parents, or controlling and punitive everyday relationships—may weaken self-control and, in turn, escalate problematic behaviour.

Dispositional traits are also relevant: conscientiousness and agreeableness relate positively, and neuroticism negatively, to self-control (58–60). Although a number of personality and environmental correlates of self-control have thus been identified, the way impulsivity in particular shapes self-control is not well understood.

Glenn and Klonsky (22) reported that the impulse-control of individuals who self-injure is poorer specifically under negative affect, while being comparable to that of others otherwise, and called for studies examining self-injurers’ performance on impulsivity tasks under different mood states. Building on this, we propose Hypothesis 3 (H3): impulsivity negatively predicts self-control in senior high school students.

Integrating these strands, the affect-regulation model of NSSI (61, 62) holds that NSSI primarily functions to relieve negative emotions, while the self-control strength model (48, 63) holds that self-control draws on a limited reservoir of psychological resources. Because impulsive individuals tend to lack effective self-regulation, highly impulsive adolescents may have fewer psychological resources available for self-control and therefore be more prone to NSSI. The mechanism linking these constructs has rarely been tested directly. We therefore propose Hypothesis 4 (H4): self-control mediates the relationship between impulsivity and NSSI.

### Left-behind experience as a moderating early-life adversity

The family environment is a further critical influence on NSSI, with an adverse environment acting as a key risk factor (64, 65). China has up to 200 million people with a left-behind experience, many of whom have now entered high school and university. Following Jin (66), left-behind students are defined here as those whose parent(s) migrated for work and left them in their hometown, with the separation beginning before 14 years of age and lasting at least half a year on each occasion. Existing research has largely documented the current psychological status of left-behind children, showing that the experience can affect mental health and exert long-term effects; far less is known about how it shapes later development or about possible protective mechanisms. From a developmental perspective, the present study therefore asks whether the mechanism underlying NSSI in late adolescence differs between individuals with and without a left-behind experience.

**Figure 1:**
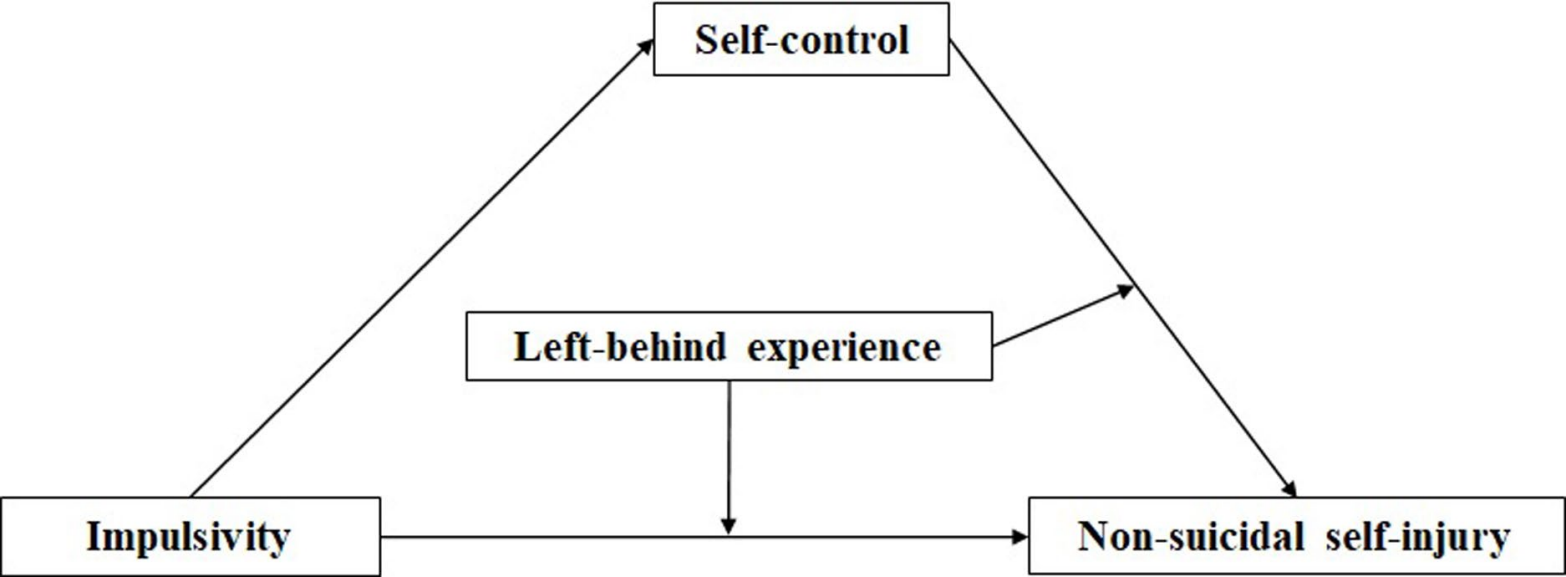
Hypothesised model of the relationship among impulsivity, non-suicidal self-injury, self-control and left-behind experience among high school students.

Nock’s integrated theoretical model (14) proposes that distal causal agents—including genetic factors, early experience, and education—jointly influence NSSI. Within this framework, a left-behind experience constitutes a salient early experience that may combine with dispositional and self-regulatory factors (impulsivity and self-control) to shape NSSI, so that the specific pathway to NSSI may differ for adolescents with versus without such an experience. Accordingly, we propose Hypothesis 5 (H5): left-behind experience moderates the mechanism through which impulsivity and self-control relate to NSSI in senior high school students (see Figure 1).

### The present study

This integrated approach also responds to a known limitation of the NSSI literature. A meta-analysis of 20 studies found that, although many statistically significant risk factors have been identified (e.g., a history of self-injury and hopelessness), their combined predictive power is low, with an overall weighted mean odds ratio of 1.56 that falls to 1.16 after correction for publication bias (67). NSSI is a complex behaviour shaped by multiple, potentially interacting determinants, yet the literature still offers limited evidence on how nature and nurture interact to produce it. Examining impulsivity, self-control, and left-behind experience together—and within the Chinese senior-high-school context, where conflicts between external norms and internal dispositions are especially salient—is intended to provide a more integrated and theoretically grounded account of NSSI risk.

Taken together, three features distinguish the present study from previous work. (a) What previous studies have done: prior research has typically linked impulsivity, self-control, or adverse early experiences to NSSI one factor at a time—for example, treating impulsivity as a direct predictor of NSSI, or documenting that left-behind children show poorer mental-health outcomes—and has rarely integrated a dispositional vulnerability and a self-regulatory resource within one model in non-clinical adolescents. (b) What the present study does: guided by Nock’s (14) integrated theoretical model and the self-control strength model (48), we specify and test a single moderated-mediation model in which self-control mediates the impulsivity–NSSI association and early left-behind experience moderates this mediated pathway, using a community sample of Chinese senior high school students. (c) What is different and novel: rather than asking whether left-behind experience predicts NSSI directly, we ask whether it changes the mechanism through which impulsivity is linked to NSSI—that is, whether the same dispositional risk reaches NSSI through different routes for adolescents with versus without an early history of parent–child separation. To our knowledge, this conditional-mechanism question has not previously been examined in this population, and answering it is the central theoretical and empirical contribution of the study.

## Methods

### Participants and methodology

Participants were recruited through class-based cluster sampling, with intact classes serving as the sampling unit. Two ordinary public senior high schools located in remote counties of Guizhou Province—a less-developed inland province in southwest China—took part in the study. The students were predominantly from rural family backgrounds, with a smaller proportion from working-class families, which is broadly representative of the senior high school population in remote counties of this region. Nine intact classes were sampled across the three grades (Grades 10–12) of the two schools, and all students present in the selected classes on the day of administration were invited to participate. Trained research assistants—that is, the test administrators, not the participants—administered the paper-and-pencil questionnaires collectively in the respective classrooms. Before the survey, the administrators explained the purpose of the study and the principles of voluntariness, anonymity, and confidentiality; written informed consent was obtained from all participating students, and for participants under 18 years of age parental or legal-guardian consent was additionally obtained in advance. Participants completed the questionnaires independently and according to their actual circumstances within the allotted class time. Of the 564 questionnaires distributed, 31 with missing data or invariant (careless) response patterns were excluded, leaving 534 valid questionnaires (male = 209, female = 325; valid response rate = 94.5%). Participants were drawn from Grade 10 (*N* = 182), Grade 11 (*N* = 177), and Grade 12 (*N* = 175), with a mean age of 17.26 years (SD = 1.30). Of these, 266 students were classified as having a left-behind experience and 268 as not (see the following section for the classification criteria).

### Measurement of left-behind experience

Left-behind experience was assessed with three self-report items adapted from prior research on Chinese left-behind children (66). The first item assessed parental migration: “Since you were born, have your parents gone out to work as migrant workers?” (1 = father working away, 2 = mother working away, 3 = both parents working away, 4 = both parents at home). The second item assessed the duration of separation: “For how many years have your parent(s) worked away from home?” (when the two parents differed, the longer duration was recorded; 1 = half a year to 1 year; 2 = 1–2 years; 3 = 2–5 years; 4 = more than 5 years). The third item assessed the age at which the separation began: “At what age did your parent(s) begin to work away from home?” (1 = 1–3 years, 2 = 3–6 years, 3 = 6–12 years, 4 = 12–15 years). On the basis of these three items, and following the operational definition of left-behind students (66), a participant was classified as having a left-behind experience (coded 1) when all of the following conditions were met: (a) at least one parent had worked away from home; (b) the cumulative duration of parent–child separation was at least half a year; and (c) the separation began before 14 years of age. Participants who did not meet these criteria were classified as not having a left-behind experience (coded 0). This procedure yielded 266 participants with a left-behind experience and 268 without.

### Research instruments

Barratt Impulsiveness Scale (BIS-11). Impulsivity was measured with the Chinese version of the Barratt Impulsiveness Scale (BIS-11) (27), translated and adapted by the Beijing Suicide Research and Prevention Centre. The adapted scale comprises 30 items across three dimensions (motor, cognitive, and non-planning impulsivity) and has demonstrated good reliability and validity for research and clinical use in China (68). Items are rated on a 5-point Likert scale (1 = never, 5 = always), with the non-planning and cognitive impulsivity items reverse-scored. Item scores were transformed to derive the sub-scale scores, with higher scores indicating stronger impulsivity. Chinese norms for high school students have been established for this scale, and its application in high school samples has shown satisfactory reliability and validity (69). Cronbach’s alpha in the present sample was 0.856.

Self-control Scale for High School Students. Self-control was measured with the scale developed by Wang and Lu (30), which comprises three dimensions: emotional self-control (e.g., “I get very annoyed when I cannot figure out a difficult question”), behavioural self-control (e.g., “Once I start playing video games, I cannot stop”), and cognitive self-control (e.g., “I am often absent-minded in class”). Of the 36 items, 10 are scored normally and 26 are reverse-scored, on a 5-point Likert scale (1 = totally disagree, 5 = totally agree). Higher total scores reflect better self-control. Cronbach’s alpha in the present sample was 0.825.

We used the behavioural scale of the localised NSSI Assessment Questionnaire suitable for Chinese adolescents, developed by Wan. et al. (70) to conduct our survey. The questionnaire lists 12 specific self-injurious behaviours and comprises two dimensions: (1) self-injury without evident tissue damage (e.g., pinching or scratching oneself, pulling one’s own hair) and (2) self-injury with evident tissue damage (e.g., cutting or burning the skin, or carving words or symbols into it). For each behaviour, participants reported how frequently they had engaged in it on a 5-point Likert frequency scale (0 = never, 1 = occasionally, 2 = sometimes, 3 = often, 4 = always). A total NSSI score was computed by summing the 12 items, with higher scores reflecting a higher overall frequency of NSSI engagement; the score therefore indexes the frequency of NSSI rather than its mere lifetime presence or its clinical severity. The Cronbach’s alpha of the scale in the present sample was 0.930.

### Data analysis

All analyses were conducted with SPSS 26.0 (IBM SPSS Statistics) and the PROCESS macro (71), with gender and age included as covariates throughout. Pearson correlations were first computed among all study variables. A hypothesis-driven, two-step analytic strategy was then adopted, following the conceptual logic of the proposed model. In the first step, the simple mediation hypothesis (H4) was tested using PROCESS Model 4 in order to establish whether self-control mediates the impulsivity–NSSI association. In the second step, given that the mediation pathway was supported, the moderated-mediation hypothesis (H5) was tested using PROCESS Model 15, which estimates, within a single integrated model, the moderation by left-behind experience of both the direct path and the second stage of the indirect path. This sequential procedure places the simple mediation model and the moderated-mediation model within one coherent analytical framework while keeping the test of each hypothesis transparent. All indirect, conditional, and index-of-moderated-mediation effects were estimated using bias-corrected bootstrapping with 5,000 resamples and 95% confidence intervals (CIs); an effect was considered significant when its CI excluded zero. Because participants were sampled in intact classes, the data have a nested (clustered) structure; the implications of this design for the precision of the estimates are addressed in the Limitations section.

### Common method bias testing

All questionnaires were completed anonymously, several items were reverse-worded, and procedural remedies were applied during administration to limit potential common method bias. Harman’s single-factor test (72) identified 19 factors with eigenvalues greater than 1, and the largest single factor accounted for 14.79% of the total variance, well below the conventional 40% threshold. To evaluate common method variance further, a single-factor confirmatory model was specified in which all impulsivity, self-control, and NSSI items were forced to load on one common latent factor. This model fitted the data very poorly (χ^2^/df = 35.05, CFI = 0.606, TLI = 0.291, RMSEA = 0.253). It should be emphasised that this test does not assess the construct validity of the individual scales: in this analysis a poor fit is the desired outcome, because it indicates that items from the three different constructs cannot be adequately represented by a single underlying method factor. Taken together with Harman’s test, these results suggest that common method variance is unlikely to distort the present findings seriously. Nevertheless, because all variables were assessed by self-report at a single time point, common method variance cannot be fully ruled out; this caveat is revisited in the Limitations section.

## Results

### Descriptive statistics and correlation coefficients of variables

Descriptive statistics and correlations were computed as an initial assessment of the relations among impulsivity, self-control, NSSI, and left-behind experience (Table 1). Impulsivity correlated positively with NSSI and negatively with self-control, and self-control correlated negatively with NSSI. In other words, students higher in impulsivity reported more NSSI and lower self-control, and students higher in self-control reported less NSSI. Left-behind experience was not significantly correlated with impulsivity, self-control, or NSSI.

**Table 1:** Descriptive statistics and correlation coefficients of variables.

Variable	Sex	Age	Impulsivity	Self-control	NSSI
Sex	1				
Age	−0.010	1			
Impulsivity	0.156**	0.037	1		
Self-control	−0.066	0.013	−0.556***	1	
NSSI	−0.089*	−0.015	0.225***	−0.251***	1
Left-behind experience	−0.007	−0.035	−0.063	0.039	0.038
M	-	17.260	44.524	112.043	4.715
SD	-	1.299	11.845	16.981	7.603

*N* = 534. Sex was coded 1 = male, 2 = female; left-behind experience was coded 0 = without, 1 = with. NSSI, non-suicidal self-injury; M, mean; SD, standard deviation. *p < 0.05, **p < 0.01, ***p < 0.001 (two-tailed).

### Testing for the mediation effect

Mediation was tested using Model 4 of the PROCESS macro for SPSS (Table 2). Impulsivity alone significantly and positively predicted NSSI, and significantly and negatively predicted self-control. When self-control was entered as a mediator, impulsivity continued to predict NSSI positively, and self-control predicted NSSI negatively. The bias-corrected percentile bootstrap test confirmed a significant indirect effect through self-control (indirect effect = 0.100, 95% CI [0.040, 0.170]), accounting for 40.82% of the total effect (0.245). Thus, impulsivity was related to NSSI both directly and indirectly through its association with self-control.

**Table 2:** Results of mediated regression analyses.

Predictors	Model 1: NSSI (dependent variable)			Model 2: Self-control (dependent variable)			Model 3: NSSI (dependent variable)		
	*b*	SE	*t*	*b*	SE	*t*	*b*	SE	*t*
Sex	−0.258	0.087	−2.948	0.046	0.075	0.613	−0.250	0.087	−2.885**
Age	−0.023	0.033	−0.698	0.027	0.028	0.946	−0.018	0.032	−0.559
Impulsivity	0.245	0.043	5.755^***^	−0.560	0.037	−15.314^***^	0.146	0.051	2.873^**^
Self-control							−0.178	0.050	−3.544^***^
*R* ^2^	0.067			0.311			0.088		
F	12.535^***^			79.331^***^			12.747^***^		

*N* = 534. All continuous predictors were standardised before model estimation; sex and age were entered as covariates. b, unstandardised regression coefficient; SE, standard error; NSSI, non-suicidal self-injury. *p < 0.05, **p < 0.01, ***p < 0.001.

### Testing for moderated mediation

It should be noted that the inclusion of left-behind experience as a moderator was theory-driven rather than data-driven. Although Table 1 shows that the zero-order correlations between left-behind experience and impulsivity, self-control, and NSSI did not reach statistical significance, the absence of a significant first-order correlation does not preclude an interaction effect; on the contrary, contemporary methodological guidance explicitly cautions against using zero-order correlations as a screening criterion for testing moderation (71). Nock’s (14) integrated theoretical model of NSSI and Bronfenbrenner’s ecological framework both argue that early family experiences shape how individual susceptibility (e.g., impulsivity) and self-regulatory capacity (e.g., self-control) translate into self-injurious behaviour. On these theoretical grounds—and consistent with H5, which was specified *a priori*—left-behind experience was tested as a moderator of the impulsivity–self-control–NSSI pathway rather than as an additional direct predictor.

Moderated mediation was tested using Model 15 of the PROCESS macro (71), which allows left-behind experience to moderate both the direct path from impulsivity to NSSI and the second stage of the indirect path (from self-control to NSSI). As shown in Table 3, the Impulsivity × Left-behind experience interaction (95% CI [−0.403, −0.007]) and the Self-control × Left-behind experience interaction (95% CI [−0.418, −0.024]) both significantly predicted NSSI. The index of moderated mediation was 0.124 (95% CI [0.010, 0.250]), confirming that the mediated pathway was significantly moderated by left-behind experience.

**Table 3:** Results of moderated mediation regression analyses.

Predictors	NSSI (dependent variable)		
	*b*	SE	*t*
Sex	−0.245	0.086	−2.839**
Age	−0.015	0.032	−0.456
Impulsivity	0.466	0.164	2.836**
Self-control	0.161	0.162	0.997
Left-behind experience	0.104	0.083	1.243
Impulsivity* Left-behind experience	−0.205	0.101	−2.036*
Self-control * Left-behind experience	−0.221	0.100	−2.206*
*R* ^2^	0.101		
F	8.391***		

*N* = 534. All continuous predictors were standardised before model estimation; sex and age were entered as covariates. Left-behind experience was coded 0 = without, 1 = with. b, unstandardised regression coefficient; SE, standard error; NSSI, non-suicidal self-injury. * *p* < 0.05, ** *p* < 0.01, *** *p* < 0.001.

Bias-corrected percentile bootstrap analyses further indicated that the indirect relation between impulsivity and NSSI via self-control was moderated by left-behind experience. Among students with a left-behind experience, the indirect effect was significant (indirect effect = 0.157, 95% CI [0.064, 0.263]) whereas the direct effect was not (direct effect = 0.056, 95% CI [−0.077, 0.189]). Among students without a left-behind experience, the pattern was reversed: the indirect effect through self-control was not significant (indirect effect = 0.034, 95% CI [−0.031, 0.098]) but the direct effect was (direct effect = 0.261, 95% CI [0.113, 0.409]). Thus, for students with a left-behind experience, NSSI was linked to impulsivity mainly through the indirect impulsivity–self-control–NSSI pathway, whereas for students without such an experience the association was predominantly direct.

**Figure 2:**
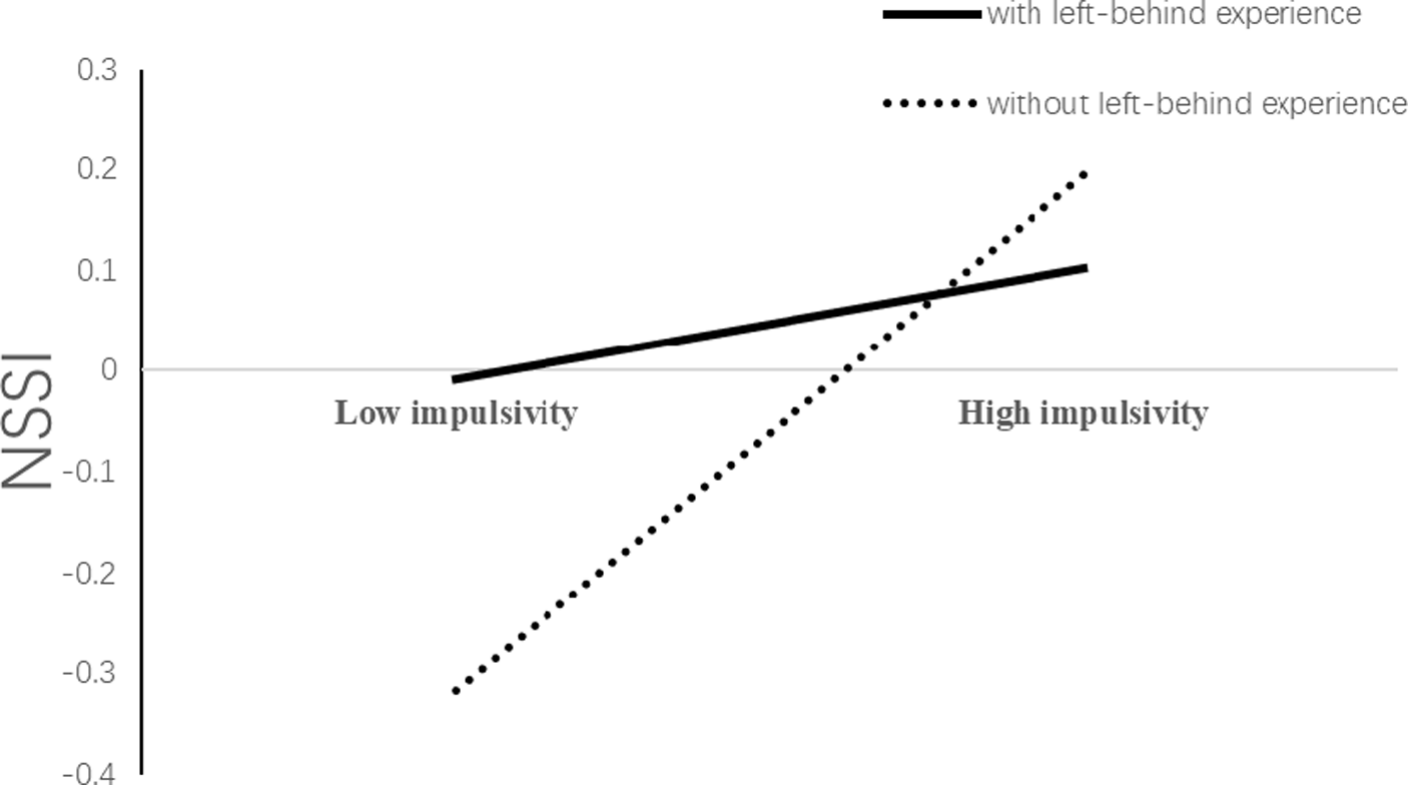
Left-behind experience moderates the effect of impulsivity on NSSI.

Predicted NSSI was plotted against impulsivity separately for students with and without a left-behind experience (Figure 2). Simple slope tests showed a significant positive association between impulsivity and NSSI for students without a left-behind experience (effect = 0.261, 95% CI [0.113, 0.409]), whereas this association was non-significant for students with a left-behind experience (effect = 0.056, 95% CI [−0.077, 0.189]).

**Figure 3:**
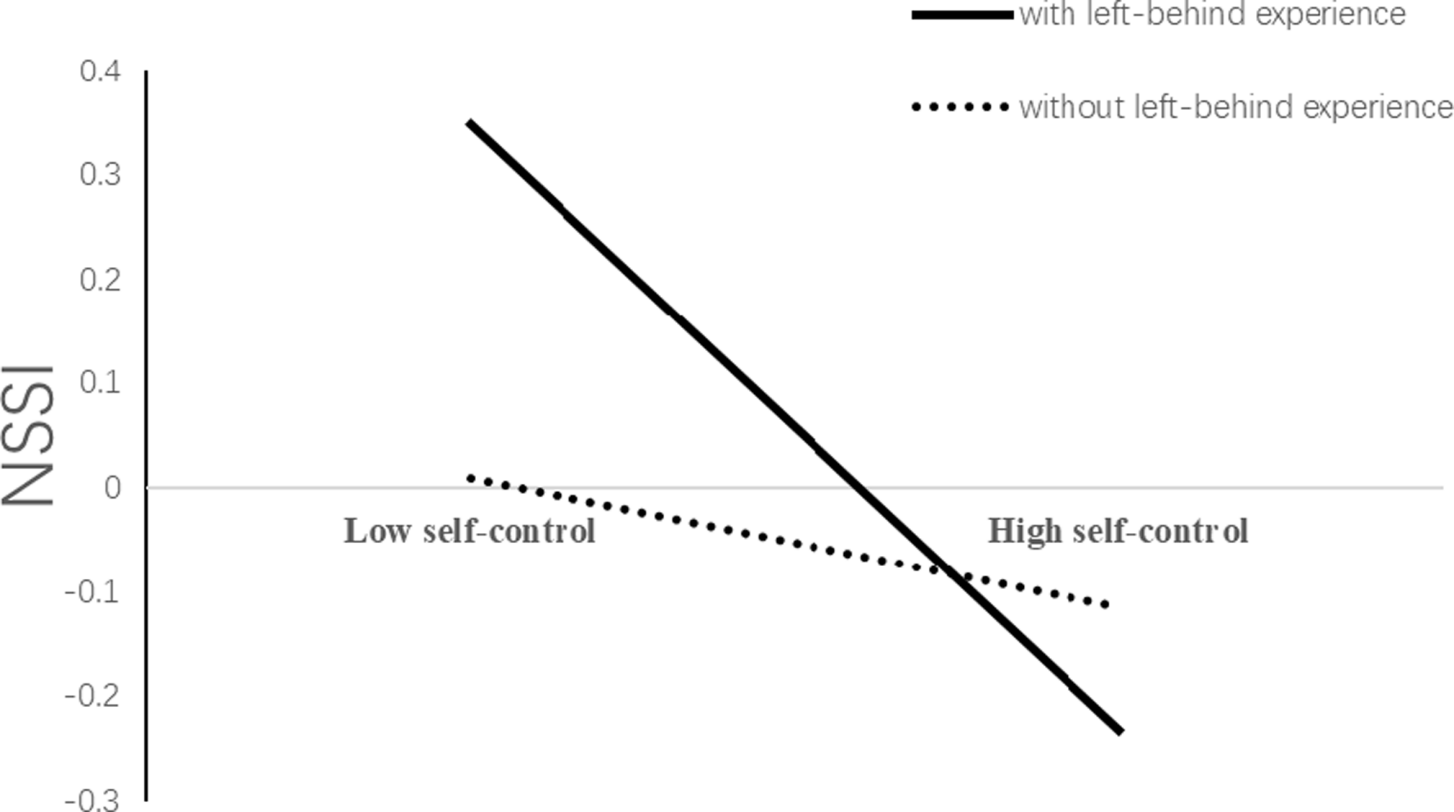
Left-behind experience moderates the effect of self-control on NSSI.

Predicted NSSI was also plotted against self-control separately for students with and without a left-behind experience (Figure 3). Simple slope tests showed a significant negative association between self-control and NSSI for students with a left-behind experience (effect = −0.281, 95% CI [−0.416, −0.147]), whereas this association was non-significant for students without such an experience (effect = −0.060, 95% CI [−0.204, 0.084]).

## Discussion

This study examined the relationship between impulsivity and NSSI among senior high school students in China, testing the mediating role of self-control and the moderating role of left-behind experience within a single integrated model. The findings generally supported our hypotheses: impulsivity was significantly associated with NSSI, self-control mediated this association, and left-behind experience moderated the mediated pathway.

Consistent with Hypothesis 1, students higher in impulsivity were more prone to NSSI, in line with prior evidence linking impulsivity to poorer mental health in children and adolescents (24–26) and to suicide attempts (73).

The transition from childhood to adulthood involves substantial psychological, physiological, and social changes (74, 75). Impulsivity peaks between roughly 13 and 17 years of age and reliably predicts adolescent misconduct (76, 77), and it has been identified as a predictor of both the onset and the maintenance of self-injurious behaviour in adolescence (78). Relatedly, a Chinese study of the Big Five traits and NSSI in high school students found that highly neurotic individuals were more likely to engage in repeated NSSI (79); neuroticism shares considerable ground with high impulsivity. Adolescents with this profile tend to view problems in absolute, one-sided terms, struggle to regulate their behaviour, magnify minor everyday difficulties, and plan poorly for the future. When they cannot find constructive solutions to their problems, NSSI may become a maladaptive source of relief.

Highly impulsive individuals also tend to freeze in the face of emergencies and negative events and to be less sensitive to negative outcomes; impulsive students may therefore cope poorly with negative life events, increasing NSSI and suicide risk (80). Our results further showed that students with lower self-control reported more NSSI and vice versa, supporting Hypothesis 2 and echoing the literature on self-control and adolescent misconduct. According to problem-behaviour theory, weak self-control is a key psychological mechanism underlying delinquent conduct (81); it predicts antisocial and rule-breaking behaviour (36) as well as smoking, drug use, and alcohol abuse (46, 82). In addition, individuals with low self-control often lack emotion-regulation skills, which leaves them more exposed to intense negative emotions and less able to distance themselves from them, thereby raising NSSI risk. Notably, self-control training has been found to reduce aggression in aggressive individuals (82), pointing to the malleability of this capacity.

According to the self-control strength model, high school students with stronger self-control have more psychological resources available, which provides additional positive psychological energy when facing emotional disturbances or other problems. This may help them to align their behaviour with social or personal standards, thereby reducing the likelihood of NSSI. Consistent with this account, our mediation model showed that self-control statistically mediated a substantial portion of the association between impulsivity and NSSI, suggesting that impulsivity is an important correlate of NSSI and that part of this association is statistically accounted for by lower self-control. These patterns are consistent with H3 and H4. Because the present design is cross-sectional, however, this mediational pattern should be interpreted as a statistical association rather than as evidence of a causal sequence; longitudinal designs are needed to establish the temporal ordering implied by the model.

Highly impulsive individuals tend to react hastily and without planning to internal and external stimuli, and often fail to manage their thoughts and behaviour effectively (20), rendering them more vulnerable to NSSI. Lacking sufficient psychological resources, they frequently fail to respond adaptively to sudden negative events and the distress these events generate, and are therefore more likely to experience persistent negative emotions (83).

A complementary account is offered by the emotion-regulation model of NSSI, which proposes that impulsive individuals, who have fewer adaptive emotion-regulation strategies, are more likely to engage in NSSI as a means of modulating intense affective states. Convergent evidence from a recent clinical study of adolescent inpatients indicated that impulsivity statistically mediated the link between childhood maltreatment and NSSI symptoms (84), while a parallel investigation identified impulsivity and emotion dysregulation as core components of a potential addictive mechanism underlying repetitive NSSI (85). These findings are consistent with our mediation pattern and indicate that, at the level of association, the impulsivity–self-control–NSSI sequence is not idiosyncratic to our sample but converges with mechanism-focused work in clinical adolescent populations. The general delinquency theory states that self-control is an important factor affecting individual problematic behaviours, and is also a key intermediate variable for other factors (such as group relations and self-esteem) to affect individual problematic behaviours (86, 87). This study also verified that self-control is an indirect variable that, along with impulsivity affect the NSSI of high school students.

The most important finding of this study concerns the moderating role of left-behind experience, which clarifies why the same dispositional risk does not operate in the same way for all adolescents. Left-behind experience significantly moderated the impulsivity–self-control–NSSI pathway, and the two subgroups showed mirror-image patterns. For adolescents with a left-behind experience, impulsivity was linked to NSSI mainly through the indirect route: higher impulsivity was associated with lower self-control, which in turn was associated with more NSSI, whereas the direct impulsivity–NSSI association was not significant. For adolescents without a left-behind experience, the reverse held: impulsivity was associated with NSSI directly, while the indirect route through self-control was not significant. Two points deserve emphasis. First, in the integrated moderated-mediation model the unconditional main effect of self-control on NSSI was not significant (b = 0.161, *p* > 0.05); the relevance of self-control was conditional, as shown by the significant self-control × left-behind experience interaction and by the significant conditional indirect effect that emerged only in the left-behind subgroup. In other words, low self-control was associated with higher NSSI specifically among adolescents who had been left behind, but added little beyond impulsivity among those who had not. Second, this pattern indicates that left-behind experience does not simply raise the overall level of NSSI; rather, it reorganises the pathway through which impulsivity reaches NSSI, shifting the dominant route from a direct dispositional effect to an indirect, self-regulatory one. Because the design is cross-sectional, these results describe how the association patterns differ between subgroups rather than a causal sequence.

By contrast, among adolescents without a left-behind experience, the indirect pathway via self-control was not significant, and impulsivity was the only significant correlate of NSSI. This conditional pattern is consistent with the theoretical model proposed by Nock (14), in which dispositional, developmental, and environmental causal agents jointly shape NSSI risk through emotion-regulation and interpersonal difficulties, and it is empirically aligned with the most recent Chinese evidence base: a meta-analysis of 165,276 individuals confirmed a 32% higher risk of NSSI in left-behind children and adolescents than in their non-left-behind peers (88), and a large-scale survey of 16,866 Chinese adolescents reported a markedly elevated NSSI incidence and lower help-seeking among left-behind children (89). Taken together with our results, these findings suggest that the impulsivity–self-control–NSSI mechanism may operate primarily in adolescents whose early family environments have weakened the development of self-regulatory resources, while in adolescents with intact family environments impulsivity may act on NSSI through other pathways such as peer- or school-based emotional triggers.

For students with a left-behind experience, parental absence occurred precisely when parental care was most needed, and the demands of the high school years then compound this early adversity, affecting mental health and development in multiple ways. A poor interpersonal environment is a key contributor to NSSI; in particular, a dysfunctional family environment is strongly implicated in emotion-regulation difficulties, leaving left-behind students less able to control themselves and more likely to engage in NSSI.

Bowlby’s attachment theory holds that consistent, secure, high-quality early experiences with parents are internalised by children as mental representations of positive attachment, laying the foundation for the stability and security needed for healthy development (90). This sense of security is strongly shaped by early caregiving (91): during childhood, attachment is expressed as reliance on, and physical closeness to, primary caregivers, and prolonged interruption of that closeness can undermine the child’s sense of security and cause lasting psychological distress (92, 93). The quality of the parent–child relationship is accordingly a key factor in the onset of NSSI among lonely left-behind adolescents (94). Our results are consistent with this account: students with a left-behind experience lacked continuous childhood attachment, which appears to exert a complex negative influence—including diminished self-control—on their psychological development. For highly impulsive students in this group, the early separation may have weakened their sense of security and, with it, their self-control, thereby increasing NSSI. By contrast, for students who grew up with their parents present, and whose development was correspondingly supported, NSSI was associated with the dispositional trait of impulsivity alone.

From the perspective of the bioecological framework of human development, the individual is embedded in a complex, multilevel system of environments, of which the family—the immediate microsystem—exerts the greatest influence (95). Families shape how individuals respond to the wider environment and, in combination with personality traits, contribute to the development of NSSI as a joint product of nature and nurture. It should also be noted that the individual-susceptibility factors implicated in NSSI are not purely innate: emotional dysregulation, impulsivity, and biological vulnerabilities all emerge from the interplay of innate endowment and acquired experience.

In sum, this study integrated a person-level susceptibility (impulsivity), an acquired self-regulatory resource (self-control), and an early-life environmental adversity (left-behind experience) within one moderated-mediation model of NSSI. Theoretically, the results support Nock’s integrated model and the self-control strength model by showing that these factors do not act in isolation but combine, and that early family environment determines which combination is at work: NSSI is best understood as the joint product of dispositional, self-regulatory, and environmental factors rather than the effect of any single cause. Practically, the finding that low self-control conveys impulsivity-related risk specifically among left-behind adolescents suggests that self-control-focused prevention may be especially valuable for this subgroup, whereas interventions for non-left-behind adolescents may need to target impulsive responding more directly.

### Future research

Several priorities for future research follow from the present findings. First, because our design is cross-sectional, the impulsivity–self-control–NSSI sequence should be re-examined using longitudinal or experience-sampling designs that can evaluate the temporal ordering of effects, as recently illustrated for the prospective pathway from impulsivity to NSSI (96). Second, the moderating role of left-behind experience should be unpacked along its constituent dimensions—timing, duration, and migration pattern—because recent reviews indicate that these features are differentially associated with adolescent mental-health outcomes (88, 89). Third, integrative models that combine person-level susceptibility (impulsivity, emotion dysregulation) with proximal contextual stressors (peer relations, school climate, problematic internet use) appear promising, as suggested by recent Chinese meta-analytic work on internet-use–NSSI associations (97). Fourth, structural equation modelling combined with multilevel analyses could be used to specify, in one confirmatory framework, the joint contribution of person- and environment-level factors while accounting for the nested structure of school-based data. Beyond these analytic priorities, the predictors of self-control under adversity are still incompletely characterised, and complementary theories of self-regulation suggest further candidate mechanisms that merit empirical scrutiny. Beyond individual susceptibility, school-level factors such as teacher support, perceived peer atmosphere, and exposure to bullying are increasingly recognised as proximal drivers of adolescent NSSI (98–100). This poses questions about the interactive relationship between impulsivity and self-control under the influence of different environmental (including school) factors, requiring more research. Here, one study incorporated the latest findings in molecular genetics and neuroscience, using the diathesis-stress model, to establish a path model for NSSI or suicidal behaviours including five (genes, nervous system, temperament characteristics, the environment, and behavioural outcomes) dimensions (101). The diathesis-stress model aims to explain how the negative environment (including child abuse, negligence, and insecure attachment) during a child’s early growth stages interacts with genetic factors, resulting in NSSI or suicidal behaviours. Therefore, under the collective influence of genetic and environmental factors, the formation mechanism of NSSI among adolescents is extremely complicated, calling for more research discussion and examination under the guidance of the latest theories.

### Limitations

Three limitations of the present study should be noted. First, the cross-sectional, single-source, self-report design precludes causal inference and does not fully rule out common method variance, despite supportive results from Harman’s test and the single-factor model. Second, the data have a class-level nested structure that was not explicitly modelled, and the sample was drawn from two senior high schools in remote counties of a single inland province (Guizhou), with a student population predominantly from rural family backgrounds, which constrains the geographic and socio-economic scope of the findings. Third, the variance explained in NSSI was modest (*R*^2^ = 0.067–0.101), and left-behind experience was operationalised as a dichotomous, retrospective indicator. With these caveats in mind, we believe that our study makes a meaningful contribution to the literature by showing that left-behind experience shapes the pathway through which impulsivity is associated with adolescent self-harm via self-control. Moreover, our study, in particular, contributes to the literature on the formation of NSSI among Chinese adolescents with left-behind experience.

## Data Availability

The original contributions presented in the study are included in the article/supplementary material, further inquiries can be directed to the corresponding author.
